# Changes in event‐based streamflow magnitude and timing after suburban development with infiltration‐based stormwater management

**DOI:** 10.1002/hyp.13593

**Published:** 2019-11-13

**Authors:** Kristina G. Hopkins, Aditi S. Bhaskar, Sean A. Woznicki, Rosemary M. Fanelli

**Affiliations:** ^1^ U.S. Geological Survey, South Atlantic Water Science Center Raleigh NC USA; ^2^ Department of Civil and Environmental Engineering Colorado State University Fort Collins CO USA; ^3^ U.S. Environmental Protection Agency, National Exposure Research Laboratory, Research Triangle Park NC USA; ^4^ U.S. Geological Survey, Maryland‐Delaware‐District of Columbia Water Science Center Baltimore MD USA; ^5^ Annis Water Resources Institute Grand Valley State University Muskegon MI

**Keywords:** best management practice, green infrastructure, hydrograph analysis, Maryland, USA, rainfall‐runoff response, stormwater, Urbanization

## Abstract

Green stormwater infrastructure implementation in urban watersheds has outpaced our understanding of practice effectiveness on streamflow response to precipitation events. Long‐term monitoring of experimental suburban watersheds in Clarksburg, Maryland, USA, provided an opportunity to examine changes in event‐based streamflow metrics in two treatment watersheds that transitioned from agriculture to suburban development with a high density of infiltration‐focused stormwater control measures (SCMs). Urban Treatment 1 has predominantly single family detached housing with 33% impervious cover and 126 SCMs. Urban Treatment 2 has a mix of single family detached and attached housing with 44% impervious cover and 219 SCMs. Differences in streamflow‐event magnitude and timing were assessed using a before‐after‐control‐reference‐impact design to compare urban treatment watersheds with a forested control and an urban control with detention‐focused SCMs. Streamflow and precipitation events were identified from 14 years of sub‐daily monitoring data with an automated approach to characterize peak streamflow, runoff yield, runoff ratio, streamflow duration, time to peak, rise rate, and precipitation depth for each event. Results indicated that streamflow magnitude and timing were altered by urbanization in the urban treatment watersheds, even with SCMs treating 100% of the impervious area. The largest hydrologic changes were observed in streamflow magnitude metrics, with greater hydrologic change in Urban Treatment 2 compared with Urban Treatment 1. Although streamflow changes were observed in both urban treatment watersheds, SCMs were able to mitigate peak flows and runoff volumes compared with the urban control. The urban control had similar impervious cover to Urban Treatment 2, but Urban Treatment 2 had more than twice the precipitation depth needed to initiate a flow response and lower median peak flow and runoff yield for events less than 20 mm. Differences in impervious cover between the Urban Treatment watersheds appeared to be a large driver of differences in streamflow response, rather than SCM density. Overall, use of infiltration‐focused SCMs implemented at a watershed‐scale did provide enhanced attenuation of peak flow and runoff volumes compared to centralized‐detention SCMs.

## Graphical abstract

Streamflow magnitude and timing were altered by suburban development, even with stormwater control measures implemented to treat 100% of the impervious area. Stormwater control measures were able to mitigate some of the impacts of increased impervious cover on peak streamflow and runoff yield, particularly for events with precipitation depths < 20 mm. Differences in impervious cover between the urban treatment watersheds appeared to be a large driver of differences in streamflow response, rather than stormwater practice density.

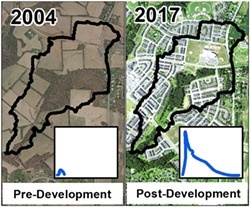

## 1 INTRODUCTION

Urban and suburban development impacts streamflow in numerous ways. Sealing the landscape surface with impervious surfaces such as roads and rooftops results in large volumes of stormwater runoff (Shuster, Bonta, Thurston, Warnemuende, & Smith, 2005). Stormwater pipe networks are constructed to manage this increased stormwater volume by routing water away from buildings and roadways and into stream channels. During the mid‐20th century, stormwater pipe networks were designed to efficiently deliver stormwater to the stream channel with little to no treatment. This type of stormwater management resulted in efficient drainage pathways causing hydrologic changes including increased frequency, volume, and magnitude of stormflow events in urban streams (Burns, Fletcher, Walsh, Ladson, & Hatt, 2012). Starting in the 1970s, communities began installing stormwater control measures (SCMs) to store and treat stormwater runoff before delivering it to streams (National Research Council, 2009). Since the 1980s, the types and locations of SCMs installed on the landscape have shifted from large, centralized SCMs focused on storing and detaining stormwater to a wide variety of smaller capacity SCMs distributed across the watershed to manage stormwater closer to where runoff is generated (Hale, 2016; McPhillips & Matsler, 2018).

The speed of SCM installation in suburban and urban watersheds has outpaced our understanding of how effective watershed‐scale installation of SCMs is for mitigating streamflow response to precipitation events. There are few empirical, watershed‐scale studies of the hydrologic impacts of installing a network of SCMs, and modelling studies may overestimate the effect of SCM treatment on stormflow response (Jefferson et al., 2017; Li, Fletcher, Duncan, & Burns, 2017). Therefore, there is a critical need for empirical monitoring to assess and validate the hydrological performance of watershed‐scale SCM implementation, rather than a reliance on individual SCM performance and modelling studies. Much of the previous empirical SCM research is based on short‐term (~1–2 years) rather than long‐term (+10 years) monitoring of urban development and SCM impacts (Li et al., 2017). Long‐term monitoring of small experimental watersheds, although notoriously hard to sustain, is fundamental to understanding streamflow change in response to environmental change, seasonal variation, and extreme flooding or drought (Tetzlaff, Carey, McNamara, Laudon, & Soulsby, 2017). Field‐based studies of streamflow generation are on the decline, but measures of hydrologic processes in headwater catchments are essential to accurately represent runoff processes in models (Burt & McDonnell, 2015).

Long‐term monitoring of experimental watersheds located in Clarksburg, Maryland, USA, provides one of the few instrumented areas where development with a high density of infiltration‐focused SCMs is implemented at the watershed‐scale and where monitoring spans before, during, and after watershed development. The U.S. Geological Survey (USGS), U.S. Environmental Protection Agency (US EPA), and Montgomery County Department of Environmental Protection (MC DEP) have monitored conditions in four study watersheds since 2004, providing over 14 years of high‐frequency (5‐minute interval) streamflow and precipitation measurements. Previous work in these watersheds has focused on analysis of aggregated flow metrics on the order of daily, monthly, or annual changes in streamflow (Bhaskar, Hogan, & Archfield, 2016; Hogan, Jarnagin, Loperfido, & Van Ness, 2014; Loperfido, Noe, Jarnagin, & Hogan, 2014) rather than sub‐daily changes in streamflow characteristics from individual storm events. These more aggregated metrics indicated that smaller flow events are better controlled by distributed SCMs than centralized SCMs (Loperfido et al., 2014), and that baseflow increased post‐development with distributed SCMs with combined loss of evapotranspiration and infiltration of stormwater (Bhaskar et al., 2016).

Examining sub‐daily streamflow responses to precipitation events provides the resolution to detect hydrologic change in small, urban watersheds with flashy and complex hydrographs, the details of which are often obscured when analyzing daily data (Horowitz, Elrick, & Smith, 2008). Analysis of sub‐daily streamflow response provides more accurate measurements of hydrologic characteristics that change rapidly within a day and are underestimated using mean daily streamflow, such as peak flows. This study capitalizes on these high frequency, long‐term datasets to answer the following questions: (1) how does urban development, coupled with a high density (> 100 SCMs/km^2^) of distributed, infiltration‐based SCMs affect streamflow response to precipitation events? (2) Do watersheds implemented with distributed, infiltration‐based SCMs exhibit similar hydrologic behaviour to a forested watershed or an urban watershed with centralized SCMs? To answer these questions, we used a before‐after‐control‐reference‐impact design to directly compare event‐based hydrograph characteristics in two suburban treatment watersheds with infiltration SCMs compared with an urban control watershed with detention SCMs and a forested control watershed.

## 2 METHODS

### 2.1 Study area

**Figure 1:**
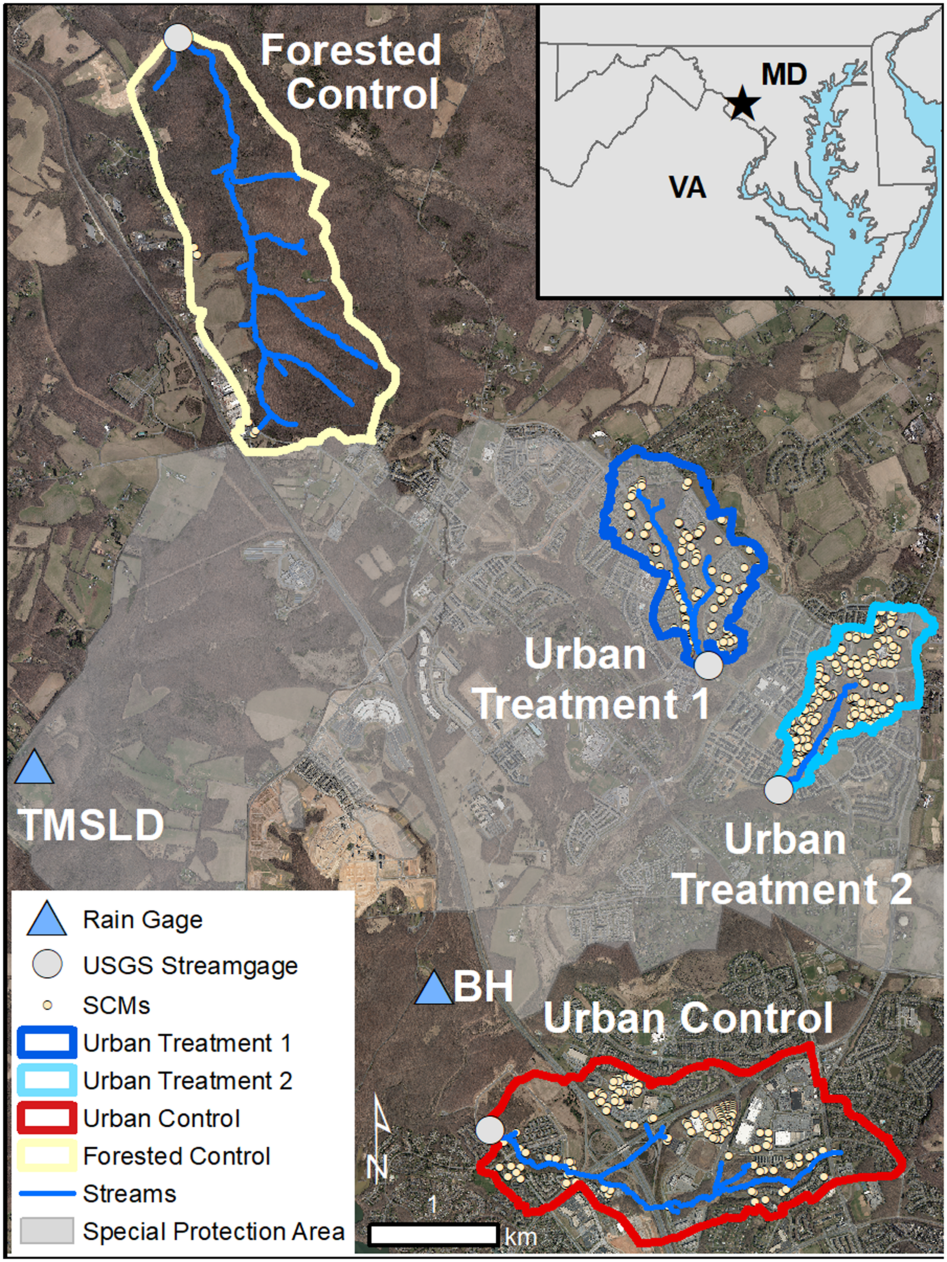
Map of the study area in Montgomery County, Maryland, with the locations of streamgages and rain gages BH (Black Hills) and TMSLD (Ten Mile Creek at Slidell, MD USGS rain gage no. 391328077185901). SCM locations within the study watersheds are shown as small circles. The gray area shows the boundary of the Clarksburg Special Protection Area (SPA). Aerial imagery from November 2011 was obtained from Montgomery County Department of Planning

This study is located in Montgomery County, Maryland, USA, within the Clarksburg Special Protection Area (39°13'51"N, 77°15'22"W), a portion of Montgomery County with high‐quality or unusually sensitive water resources and where water resources are threatened by land use changes (MC DEP, 1994) (Figure 1). Regulations in Montgomery County require that new and expanded development projects within the Special Protection Area include SCMs to mitigate the impacts of stormwater runoff on downstream water quality and quantity. Developers are required to install temporary sediment and erosion control structures during construction and utilize environmental site design to the maximum extent possible to target replicating the hydrology of “woods in good condition” (MC DEP, 2001; MDE, 2000). Environmental site design attempts to mitigate the impacts of new urban development by installing small‐scale SCMs to reduce runoff, increase infiltration, minimize impervious cover, and conserve natural features like slopes, soils, and forests (MDE, 2000). Infiltration‐focused SCMs (e.g., sand filter, bioretention) are typically designed to mitigate a 1‐inch (25.4 mm) storm depth, whereas detention ponds which serve as the last line of stormwater treatment are sized to control the 1‐year 24‐hour storm event (66 mm of precipitation), based on Maryland Department of the Environment stormwater standards (MDE, 2000).

Four study watersheds were monitored: two watersheds that underwent suburban development with a high density of SCMs, one urban control already implemented with primarily detention SCMs, and one forested control (Figure 1, Table 1). All watersheds are in the crystalline Piedmont underlain by loam and silt loam soils with moderate to low infiltration rates. The Maryland Piedmont has a legacy of agriculture that resulted in land clearing to grow agricultural crops, subsequent erosion due to poor farming practices, followed by farm abandonment and the regrowth of secondary forest post‐1930s (Jacobson & Coleman, 1986). This agricultural legacy is evident in solute export within the Clarksburg Special Protection Area and the watersheds examined in this study (Hopkins, Loperfido, Craig, Noe, & Hogan, 2017). The climate normal from 1981–2010 at Damascus, MD (USC00182336), roughly 5 km from the study area, indicates mean annual precipitation is 1178 mm and mean annual temperature is 11.9 degrees Celsius (Arguez et al., 2012). Mean annual precipitation during the study period was 1212 mm, 34 mm above the 20‐year normal (Table S1 in the Supporting Information). Water years 2014 and 2018 were the two wettest years with precipitation greater than 1430 mm and water years 2005 and 2017 were the driest years in the study period with precipitation less than 1000 mm (Table S1). The water year represents conditions from October 1st of one year through September 30th of the next year, with the year designated as the calendar year that it ends in (e.g., water year 2005 includes October 1, 2004 through September 30, 2005).

**Table 1:** Watershed characteristics for each monitoring location. Era of watershed development was determined from parcel‐level property tax assessment records from Montgomery County Department of Planning and barren land use. Density of SCMs was based on data provided by Montgomery County Department of Environmental Protection representing SCMs as of February 2017

Watershed Name	Watershed Description	USGS Streamgage Number	Watershed Area (km^2^)	Impervious Cover in 2017 (%)	Era of Watershed Development	Primary Types of SCMs	SCM Density no/km^2^	Swale Density km/km^2^ (Swale Length)
Forested control	Forested reference	01643395	3.4	2%	NA	NA	2 (*n* = 5)	No data
Urban control	Suburban and commercial with detention‐based SCMs	01644375	3.1	40%	1983–1990	Detention ponds	47 (*n* = 144)	No data
Urban Treatment 1	Suburban with infiltration‐based SCMs	01644371	1.2	33%	2004–2010	Recharge chambers and infiltration trenches	105 (*n* = 126)	4.3 (5.2 km)
Urban Treatment 2	Suburban with infiltration‐based SCMs	01644372	0.8	44%	2008–2014	Tree boxes, infiltration trenches, and underground detention	274 (*n* = 219)	2.3 (1.8 km)

### 2.2 Land cover change and stormwater management

Land cover change was characterized in each watershed using a combination of aerial imagery and light detection and ranging (lidar) datasets. Land cover prior to 2011 was obtained from Williams et al. (2018), where land cover classification was created using heads‐up digitization. National Agricultural Imagery Program (NAIP) four‐band (red, green, blue, near‐infrared) aerial imagery at 1‐m resolution was used as the basis for land cover classification in 2011, 2015, and 2017 (Woznicki & Hopkins, 2019). The Normalized Difference Vegetation Index (NDVI) was calculated from the NAIP red and near‐infrared bands. Lidar collected in leaf‐off seasons during 2013 (for 2011 land cover) and 2017 (for 2015 and 2017 land cover) was used to derive intensity (return strength of the laser pulse at each point) and normalized digital surface model (NDSM) layers at 1‐m resolution. Finally, an impervious surface layer created by the Montgomery County Planning Department was included as ancillary data, resulting in an 8‐band stack of datasets used for classification (red, green, blue, near‐infrared, NDVI, intensity, NDSM, and impervious). Classifications were completed using pixel‐based supervised classification with the random forest algorithm with the R caret package (Kuhn, 2008). Four classes were used: forest, grass, impervious, and soil/barren, with 20,000 training pixels classified for each class. Following classification, water was burned‐in using polygons from Montgomery County, and agricultural land use was manually identified at the field level using the 2008 Common Land Unit polygons from the U.S. Department of Agriculture (USDA) Farm Service Agency intersected with the USDA National Agricultural Statistics Service Cropland Data Layer for the appropriate year.

The soil‐barren class was used to define the construction period in the urban treatment watersheds. Years with greater than 15% barren land in the watershed were classified as the construction phase of development. The “pre‐development” monitoring period preceded the construction phase and the “post‐development” monitoring period comprised the years after construction. New housing parcel counts were also used to verify urban development periods and counts were estimated from parcel‐level property tax assessment records from Montgomery County Department of Planning.

The density and types of SCMs in each study watershed were determined from data obtained from MC DEP, representing SCMs installed as of February 2017 (Table 1 and Table S2). The two urban treatment watersheds have a high density of SCMs (>100 SCMs/km^2^) and Urban Treatment 2 has more than twice the SCM density as Urban Treatment 1 (Table 1). The majority of the SCMs in Urban Treatment 1 are stormwater recharge chambers, infiltration trenches, hydrodynamic oil‐grit separators, or sand filters (Table S2). Urban Treatment 1 has a higher density of roadside swales (4.3 km/km^2^), which replace curb and gutter, than Urban Treatment 2 (2.3 km/km^2^) (Table 1). The majority of the SCMs in Urban Treatment 2 are stormwater tree boxes, infiltration trenches, hydrodynamic oil‐grit separators, or micro‐bioretention (Table S2). SCMs in both urban treatment watersheds are installed in treatment trains with redundant runoff treatment terminating in a dry detention pond designed to mitigate peak flows for a 1 year 24‐hour event, 66 mm (2.6 inches) of precipitation (MDE, 2000). SCMs upslope of the terminal dry detention pond were typically designed to mitigate 2.54 cm (1 inch) of precipitation (MDE, 2000).

### 2.3 Precipitation data and event identification

Precipitation was obtained from two precipitation gages: 1) the Black Hills precipitation gage maintained by MC DEP and 2) the Ten Mile Creek Slidell, MD precipitation gage maintained by USGS (site: 391328077185901) (Figure 1). The Black Hills gage recorded precipitation at 5‐minute intervals and was used to characterize precipitation events from October 2004 through June 19, 2014. The Ten Mile Creek Slidell, MD gage recorded at 15‐minute intervals and was used to characterize precipitation from June 20, 2014, through September 30, 2018. Precipitation events were identified using a 6‐hour minimum inter‐event period using the Hydrological Model Assessment and Development (hydromad) R package (Andrews, Croke, & Jakeman, 2011). Total precipitation (mm), event duration (hour), and average precipitation intensity (mm/hr) were calculated for each precipitation event.

### 2.4 Streamflow data and storm event identification

Instantaneous discharge for each USGS streamgage from October 1, 2004, through September 30, 2018 was obtained from the USGS National Water Information System (USGS, 2019) using the dataRetrieval package in R (DeCicco et al., 2019). Instantaneous discharge was typically measured at 5‐minute intervals. In cases where instantaneous discharge was measured at 15‐minute intervals, linear interpolation was used to interpolate streamflow to 5‐minute increments. Time periods with streamflow gaps greater than 2 hours were identified and removed from further analysis. Construction activities in the watersheds occasionally caused artificial streamflow pulses not associated with any precipitation event. Construction pulses were evident in all watersheds except the forested control. Time periods influenced by construction activities were visually identified in the streamflow record and replaced with the minimum discharge from the prior, lagging 48‐hours and the leading 48‐hours. Construction activities were identified on no more than 5% of the total days during the monitoring period (Table 2).

**Table 2:** Summary of the number of paired precipitation and streamflow events for each watershed

Watershed	Number of precipitation events during the streamflow record	Number precipitation events with a detectable streamflow response	Number of excluded streamflow events	Percentage of record with construction activities
Forested	1214	445	23	0%
Urban Control	1031	687	34	4%
Urban Treatment 1	1284	470	31	3%
Urban Treatment 2	1199	434	32	5%

**Figure 2:**
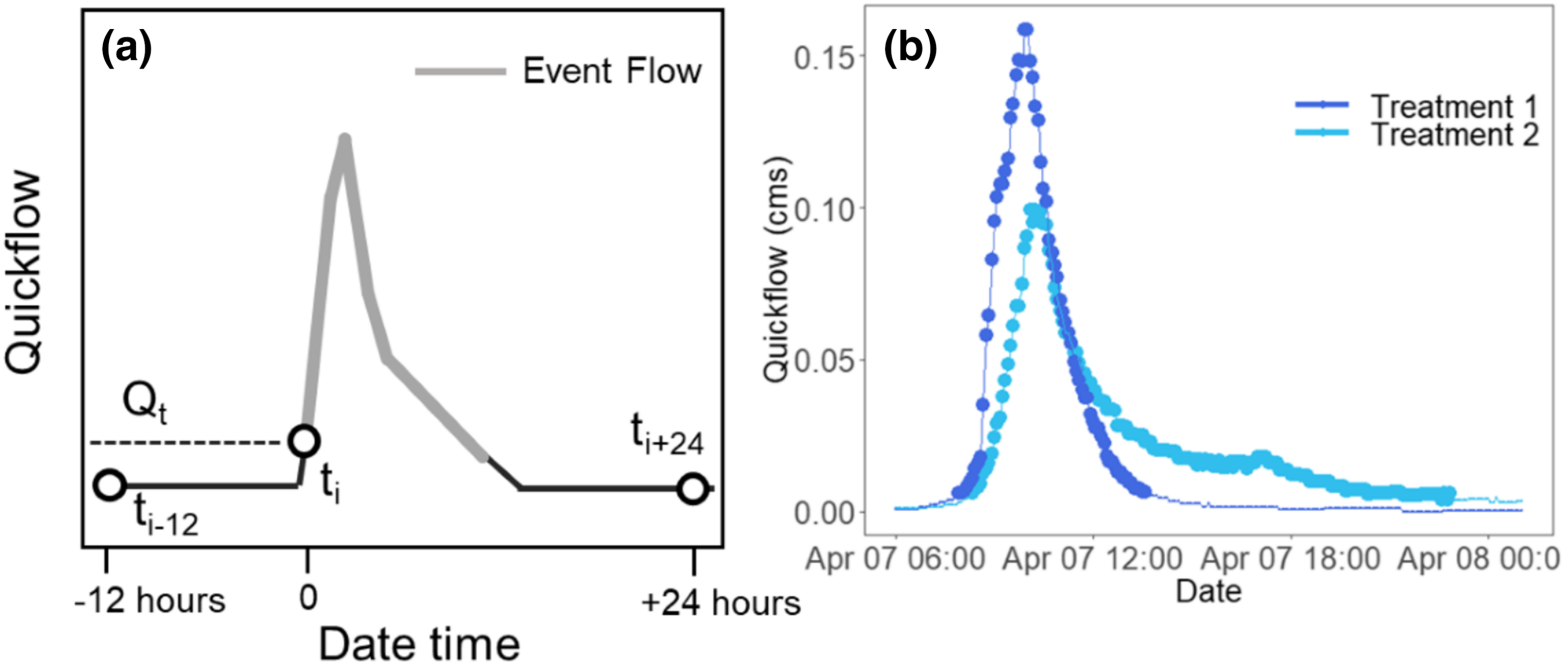
A: A streamflow event was identified using a discharge threshold (D_t_) (not shown here), a quickflow threshold (Q_t_), and two quickflow slopes calculated as quickflow at t_i_ minus the rolling 12‐hour lagging (t_i‐12_) and 24‐hr leading minimum (t_i+24_) quickflow. When this difference (t_i_ ‐ t_i‐12_ or t_i_ ‐ t_i+24_) was greater than the quickflow slope threshold (S_t_), an event was identified corresponding to criteria 3. B: Example of event identification for a 2.54 cm (1 inch) event on April 7, 2016

The vast majority of multi‐year rainfall‐runoff studies use daily‐mean discharge as opposed to instantaneous discharge. One difficulty of using instantaneous discharge is the development of an automated method for identifying streamflow responses in long‐term records. Previous researchers have used a master streamflow recession curve (Nimmo & Perkins, 2018), modified the straight line baseflow separation technique (Bell, McMillan, Clinton, & Jefferson, 2016), used a baseflow filter and a recession threshold (Duncan, 2019; Tang & Carey, 2017), or constructed dimensionless unit hydrographs (Hung, James, & Carbone, 2018). We used the BaseflowSeparation function within the EcoHydRology R package to separate the instantaneous streamflow record into quickflow and baseflow using a one parameter digital filter with a filter parameter of 0.99 and three passes (Fuka, Walter, Archibald, Steenhuis, & Easton, 2018). Previous analysis of daily mean discharge in the control watersheds and Urban Treatment 1 used a filter parameter of 0.925 (Bhaskar et al., 2016). However, adjustments to the filter parameter are necessary as the time‐step is subdivided into hourly and sub‐hourly intervals (Duncan, 2019). Therefore, a filter parameter of 0.99 was selected and validated by visually inspecting hydrographs to optimize identification of quickflow (runoff) event start and end times. Runoff events were identified using the following three criteria:

1. Discharge greater than a discharge threshold D_t_, where D_t_ was 0.057 m^3^/s (2 ft^3^/s) for watersheds with an area < 3 km^2^ and 0.085 m^3^/s (3 ft^3^/s) for watersheds with an area > 3 km^2^
2. Quickflow greater than a quickflow threshold Q_t_ of 0.007 m^3^/s (0.25 ft^3^/s)
3. Quickflow slope greater than a quickflow slope threshold S_t_ of 0.006 (0.2 ft^3^/s) calculated as quickflow minus a 24‐hour leading (t_i+24_) and 12‐hour lagging (t_i‐12_) minimum quickflow (illustrated in Figure 2)

A time increment that met any of these criteria was designated as a streamflow event (Figure 2). Streamflow events with a duration shorter than 15 minutes and that had a change in streamflow less than or equal to 0.003 cms (0.1 cfs) were excluded. A 6‐hour inter‐event window was used to define discrete streamflow events. Start and end times for streamflow and precipitation events were matched based on overlapping dates and times. This pairing was accomplished by adding 4 hours to the start of the precipitation event and 2 hours to the end of the precipitation event and identifying streamflow response start and end times within the precipitation event window. Typically, one streamflow event was matched with one precipitation event. If one streamflow event spanned two precipitation events, the metrics for those precipitation events were aggregated by summing event metrics into one event metric. If one precipitation event matched with two different streamflow events, those events were excluded from the dataset because of the complex hydrograph (Table 2).

**Figure 3:**
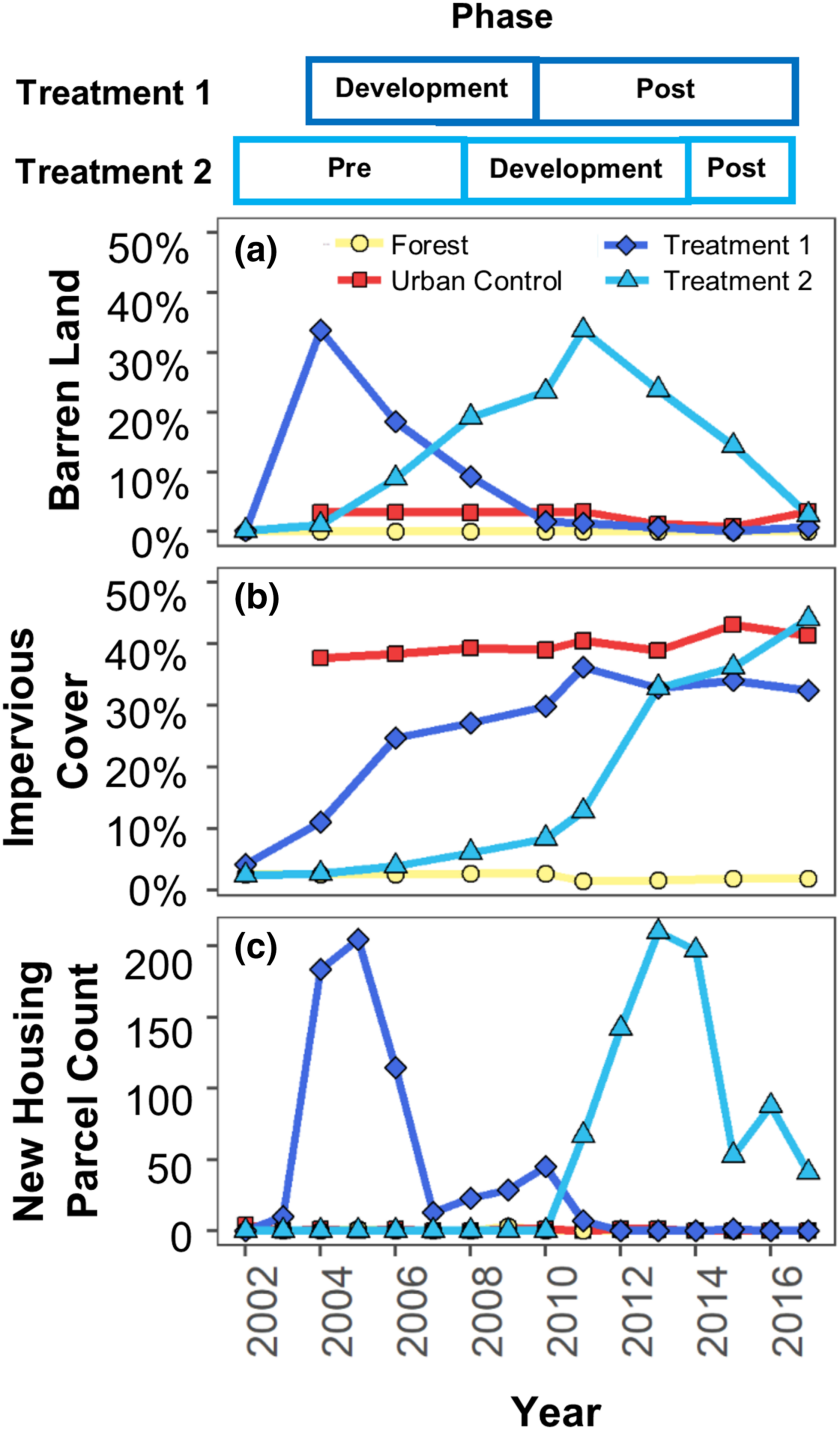
Land cover change in barren land (A), impervious cover (B), and new housing parcels (C) from 2002–2017. Pre‐development, construction, and post‐development phases for Urban Treatments 1 and 2 are shown at the top

For each streamflow event, a suite of streamflow metrics was calculated to describe characteristics important to stream ecological integrity, including magnitude, duration, timing, and rate of change (Poff et al., 1997). Three metrics were used to describe event magnitude: 1) area normalized peak streamflow, hereafter referred to as peak streamflow, 2) runoff yield calculated as quickflow volume divided by watershed area, and 3) runoff ratio calculated as runoff yield divided by precipitation depth. Three streamflow metrics were used to describe streamflow timing and rate of change: 1) event duration, 2) time to peak discharge, and 3) rise rate calculated as peak streamflow divided by time to peak discharge. Timing metrics were not compared between treatment and control watersheds because of differences in watershed size, with the control watersheds being three times larger than the treatment watersheds (~3 km^2^ compared with ~1 km^2^). Because the streamflow record in Urban Treatment 1 began during development (Figure 3), a temporal comparison of pre‐ and post‐development was limited to Urban Treatment 2.

### 2.5 Statistical analyses

All statistical analyses were performed in R Studio (R Core Team, 2018). Precipitation event thresholds were determined for the control watersheds, Urban Treatment 1 post‐development, and Urban Treatment 2 pre‐ and post‐development by developing a logistic regression model using the generalized linear model function with family set to binomial. Logistic regression models were developed using event precipitation depths and a dummy variable 1 streamflow response and 0 no streamflow response based on the runoff event criteria described above. All streamflow events that were successfully paired to a precipitation event were included (Table 2). Event precipitation depths corresponding to the 50%, 75%, and 90% probability of streamflow response were extracted to compare precipitation thresholds for each watershed and phase. The probability of streamflow response for 5‐, 10‐, and 20‐mm precipitation event were also compared.

Correlations between peak streamflow and runoff yield and event precipitation depth were assessed using simple linear regression and piecewise linear regression implemented with the segmented package (Muggeo, 2019). Variables were log transformed to meet assumptions of normality. The simple linear model was selected if the piecewise regression did not improve the R^2^ by more than 0.05. All streamflow events that were successfully paired to a precipitation event were included. Models were developed for the urban control, forested control, Urban Treatment 1 post‐development, Urban Treatment 2 pre‐development, and Urban Treatment 2 post‐development. Breakpoints of the piecewise regression and regression slopes for the second segment or simple model were compared among watersheds using the 95% confidence interval.

Comparisons of peak streamflow, runoff yield, and runoff ratios during events the urban treatment watersheds post‐development and the control watersheds were conducted by subsetting all paired events to those in which all four streamgages were functioning and no construction flows were identified. These matched streamflow responses were compared during the years 2015–2018 to correspond with the completion of watershed development in both urban treatment watersheds. Three precipitation categories were selected to roughly correspond to breakpoints from the piecewise linear regression: small (1–10 mm), medium (11–20 mm), and large (21–50 mm) events. Mean, median, and interquartile ranges for peak flow, runoff yield, and runoff ratio were calculated for each precipitation depth category. All precipitation events were analyzed whether they resulted in a streamflow response or not. Pairwise comparisons were conducted using Wilcoxon rank sum tests to assess significant differences in peak flow, runoff yield, and runoff ratios among watersheds within each precipitation depth category. Linear regression analysis was then used to assess changes in six streamflow metrics across the range of precipitation event depths (> 10 mm) pre‐ and post‐development in Urban Treatment 2.

### 2.6 Data Availability Statement

Data generated during this study are available from the USGS ScienceBase repository (Hopkins, Bhaskar, Woznicki, & Fanelli, 2019).

## 3 RESULTS

### 3.1 Land cover changes in the Treatment watersheds

Substantial land‐cover change occurred in the urban treatment watersheds during the monitoring period. Impervious cover in Urban Treatment 1 increased from 11% in 2004 to 33% in 2017, with the construction phase of development occurring from 2004–2010 (Figure 3). Impervious cover in Treatment 2 increased from 2% in 2004 to 44% in 2017, with the construction phase of development occurring from 2008–2014 (Figure 3). Montgomery County property tax records indicated peak housing construction occurred from 2004–2006 in Urban Treatment 1 and from 2012–2014 in Urban Treatment 2 (Figure 3C). Residential development during the monitoring period in Urban Treatment 1 was dominated (91% of parcels) by single‐family detached housing units (Table S3). Residential development in Urban Treatment 2 was a mix of single‐family detached (50% of parcels) and single‐family attached townhouses (50% of parcels) (Table S3). Minimal land cover change occurred in the urban and forested control watersheds during the monitoring period (Figure 3).

### 3.2 Precipitation thresholds for streamflow response

**Figure 4:**
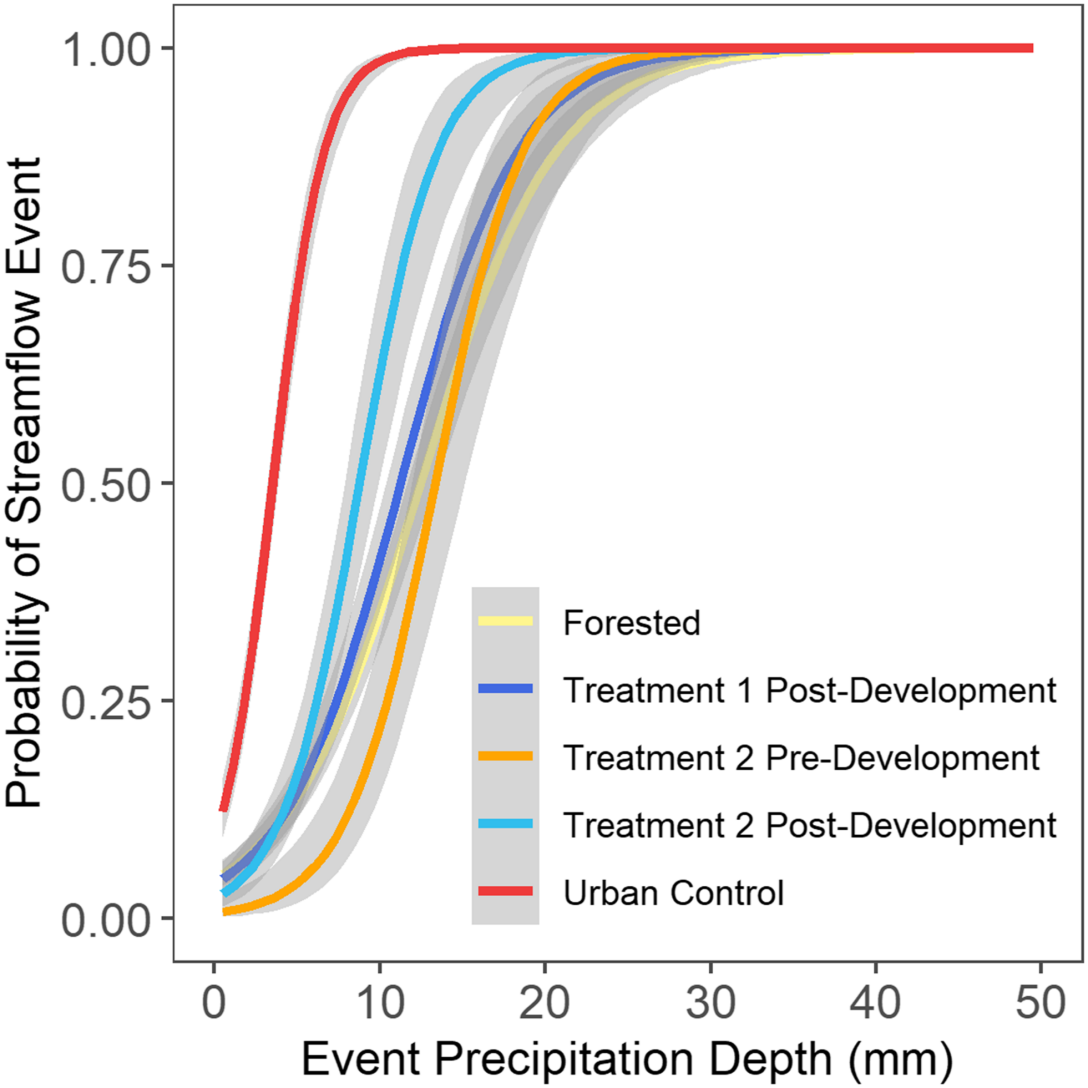
Predicted streamflow event probabilities for a range of precipitation event depths. Gray shaded area shows model standard error

Logistic regression was used to identify the probability of a streamflow response for precipitation depths up to 50 mm. The probability of a streamflow response for a 10‐mm precipitation event was lowest in Urban Treatment 2 pre‐development and highest in the urban control (Table 3). Precipitation depth needed to initiate a streamflow event was lowest in the urban control (5 mm at 75% probability) and highest in the forested control (17 mm at 75% probability), whereas Urban Treatment 1 post‐development had an intermediate value of 15.5 mm (75% probability) (Figure 4; Table 3). Precipitation amount needed to initiate a streamflow event dropped by 5 mm in Urban Treatment 2 from 16.5 mm pre‐development to 11.5 mm post‐development (75% probability, Table 3). The precipitation threshold in Urban Treatment 2 post‐development shifted toward the curve of the urban control and away from the forested site (Figure 4). The precipitation thresholds in Urban Treatment 1 post‐development (15.5 mm) and Urban Treatment 2 pre‐development (16.5 mm) were similar to the forested control (17 mm) (Figure 4, 75% probability).

**Table 3:** Model parameters and event probabilities for each watershed and phase. Akaike information criterion (AIC) is provided for each model

					Precipitation depth (mm) for given event probability			Probability of streamflow response for given precipitation depth (standard error)		
Watershed	Number of Precipitation Events	Intercept	Fitted Parameter	AIC	50%	75%	90%	5 mm	10 mm	20 mm
Forested	1,153	‐3.0*	0.24*	802.8	12.5	17	21.5	13.9% (1.2%)	35.4% (2.1%)	86.3% (2.2%)
Urban Control	969	‐2.3*	0.65*	589.7	3.5	5	7	72.4%(< 0.1%)	98.5% (< 0.1%)	100% (< 0.1%)
Urban Treatment 1 Post‐Development	730	‐3.2*	0.28*	479.4	11	15.5	19	14.6% (1.7%)	41.3% (3.0%)	92.2% (2%)
Urban Treatment 2 Pre‐Development	234	‐5.0*	0.38*	101.8	13.5	16.5	19	4.1% (1.7%)	22.1% (4.6%)	92.5% (3.9%)
Urban Treatment 2 Post‐Development	339	‐3.7*	0.43*	191.5	9	11.5	14	16.7% (2.9%)	62.8% (5.9%)	99.1% (0.1%)

* Significant at p < .01

### 3.3 Differences in peak streamflow and runoff yield across precipitation depths

**Figure 5:**
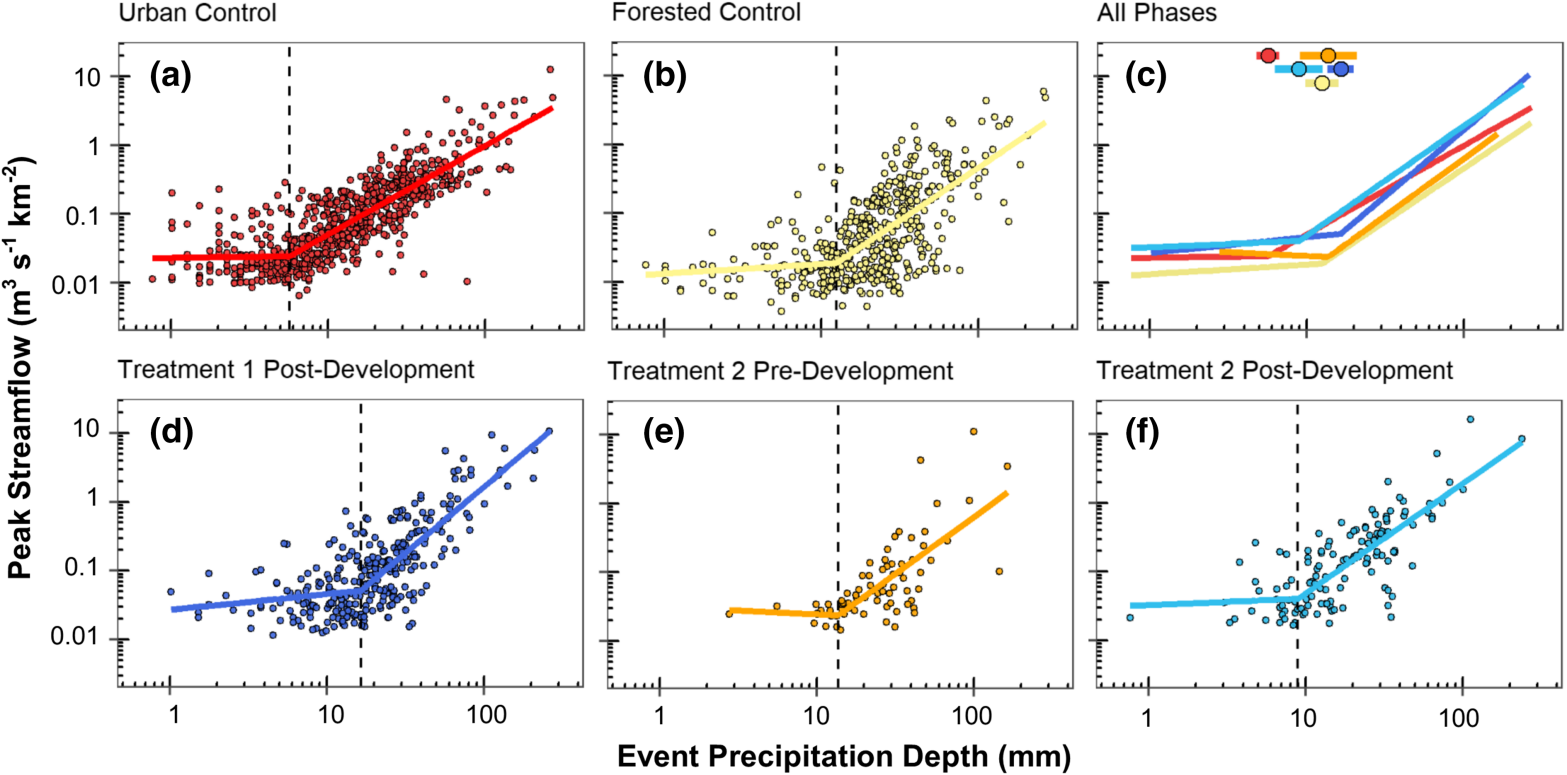
Piecewise linear regression models for peak flow versus precipitation depth in each watershed. Dotted lines indicate the breakpoint for precipitation depth. Piecewise regressions from each site are plotted together in panel C. Panel C shows breakpoint locations and 95% confidence intervals

Breakpoint analysis with log‐log linear regression was used to assess differences in peak streamflow and runoff yield across a range of precipitation depths when a streamflow response was identified. For peak streamflow, significant piecewise regression models were developed for five sites‐phase combinations. Piecewise models had higher R^2^ values than a single linear regression model for all peak streamflow models (Table 4). Peak flow increased with precipitation depth in all watersheds and phases. The precipitation depth at the breakpoint where peak flow started increasing more rapidly varied substantially among sites (Figure 5C). The urban control had the lowest breakpoint at 5.7 mm for peak flow and Urban Treatment 1 post‐development had the highest breakpoint of 16.5 mm (Table 4). Although Urban Treatment 1 post‐development had the highest peak flow breakpoint, this site also had one of the highest regression slopes indicating greater increases in peak flow with increasing precipitation above the breakpoint (Table 4). Above the breakpoints, there was higher peak flow for the same precipitation depth in the urban control, Urban Treatment 1 post‐development, and Urban Treatment 2 post‐development compared with the forested control or Urban Treatment 2 pre‐development (Figure 5C).

**Table 4:** Breakpoint model estimates for peak flow and runoff yield versus precipitation depth. All predictor and response variables were log_10_ transformed

Watershed	Piecewise linear model R^2^ (Simple linear regression R^2^)	Breakpoint (mm) (95% confidence interval)	Intercept	Slope 1 (95% confidence interval)	Slope 2 (95% confidence interval)
Peak Streamflow					
Forested	0.47 (0.38)	12.6 (9.9‐16)	‐1.87	0.14(‐0.11‐0.38)	1.54 (1.34‐1.73)
Urban Control	0.69 (0.63)	5.7 (4.8‐6.7)	‐1.64	0.03 (‐0.18‐0.24)	1.29 (1.21‐1.37)
Urban Treatment 1 Post‐Development	0.59 (0.48)	16.5 (13.6‐20.1)	‐1.56	0.22 (‐0.07‐0.51)	1.93 (1.68‐2.17)
Urban Treatment 2 Pre‐Development	0.52 (0.47)	13.8 (9.1‐20.8)	‐1.50	‐0.11(‐1.29‐1.06)	1.66 (1.24‐2.07)
Urban Treatment 2 Post‐Development	0.61 (0.56)	8.9 (6.3‐12.6)	‐1.48	0.09 (‐0.56‐0.74)	1.60 (1.32‐1.87)
Runoff Yield					
Forested†	0.50 (0.45)	NA	‐1.70	NA	1.20 (1.08‐1.32)
Urban Control†	0.74 (0.69)	NA	‐1.53	NA	1.38 (1.31‐1.45)
Urban Treatment 1 Post‐Development	0.59 (0.54)	9.8 (7.1‐13.6)	‐1.03	0.29 (‐0.30‐0.88)	1.97 (1.74‐2.20)
Urban Treatment 2 Pre‐Development†	0.48 (0.48)	NA	‐2.32	NA	1.54 (1.17–1.91)
Urban Treatment 2 Post‐Development †	0.64 (0.63)	NA	‐1.91	NA	1.65 (1.43‐1.87)

Note: † Single linear regression model parameters are reported.

**Figure 6:**
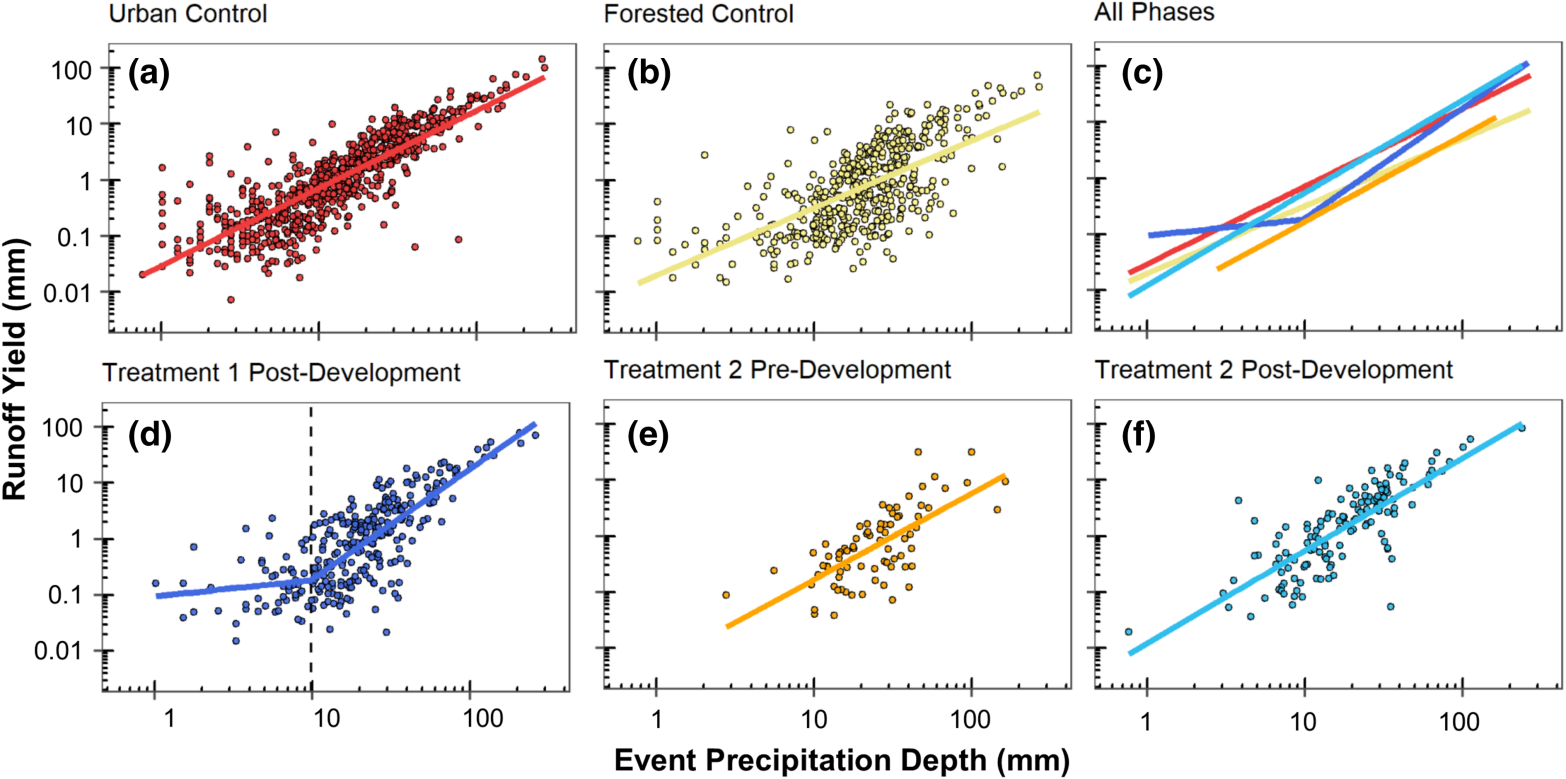
Piecewise linear regression or single linear regression for runoff yield in millimetres versus precipitation depth in each watershed. Regressions from each site are plotted together in panel C. Dotted lines indicate the breakpoint for precipitation depth

For runoff yield, simple linear regression models were developed for all phases except for Urban Treatment 1 post‐development (Table 4). Runoff yield increased with precipitation depth in all watersheds and phases (Figure 6). A breakpoint for Urban Treatment 1 post‐development was identified at a precipitation depth of 9.8 mm, below which the slope of segment 1 was not significantly different from zero (Table 4). The Urban Treatment 1 post‐development model had the greatest regression slope above the breakpoint and the forested model had the lowest regression slope (Table 4). The Urban Treatment 2 post‐development model had a slope similar to the urban control, but a lower intercept (Table 4). The peak flow and runoff yield models for the urban control were best explained by precipitation depth (highest R^2^) compared with the other sites‐phases, whereas the forested control and Urban Treatment 2 pre‐development were least explained.

### 3.4 Changes in streamflow magnitude after watershed development

Matched events were compared for the urban treatment and control watersheds during 2015 to 2018, which corresponds to post‐development in both the treatment watersheds. This time period included 172 precipitation events, with streamflow responses detected for 70%, 33%, 35% and 28% of these precipitation events in the urban control, Treatment 1 post‐development, Treatment 2 post‐development, and forested control, respectively. All 172 precipitation events were analyzed whether they resulted in a streamflow response or not across watersheds. Event peak streamflow, runoff yield, and runoff ratios were compared for three precipitation‐event categories (1–10 mm, 11–20 mm, and 21–50 mm).

**Figure 7:**
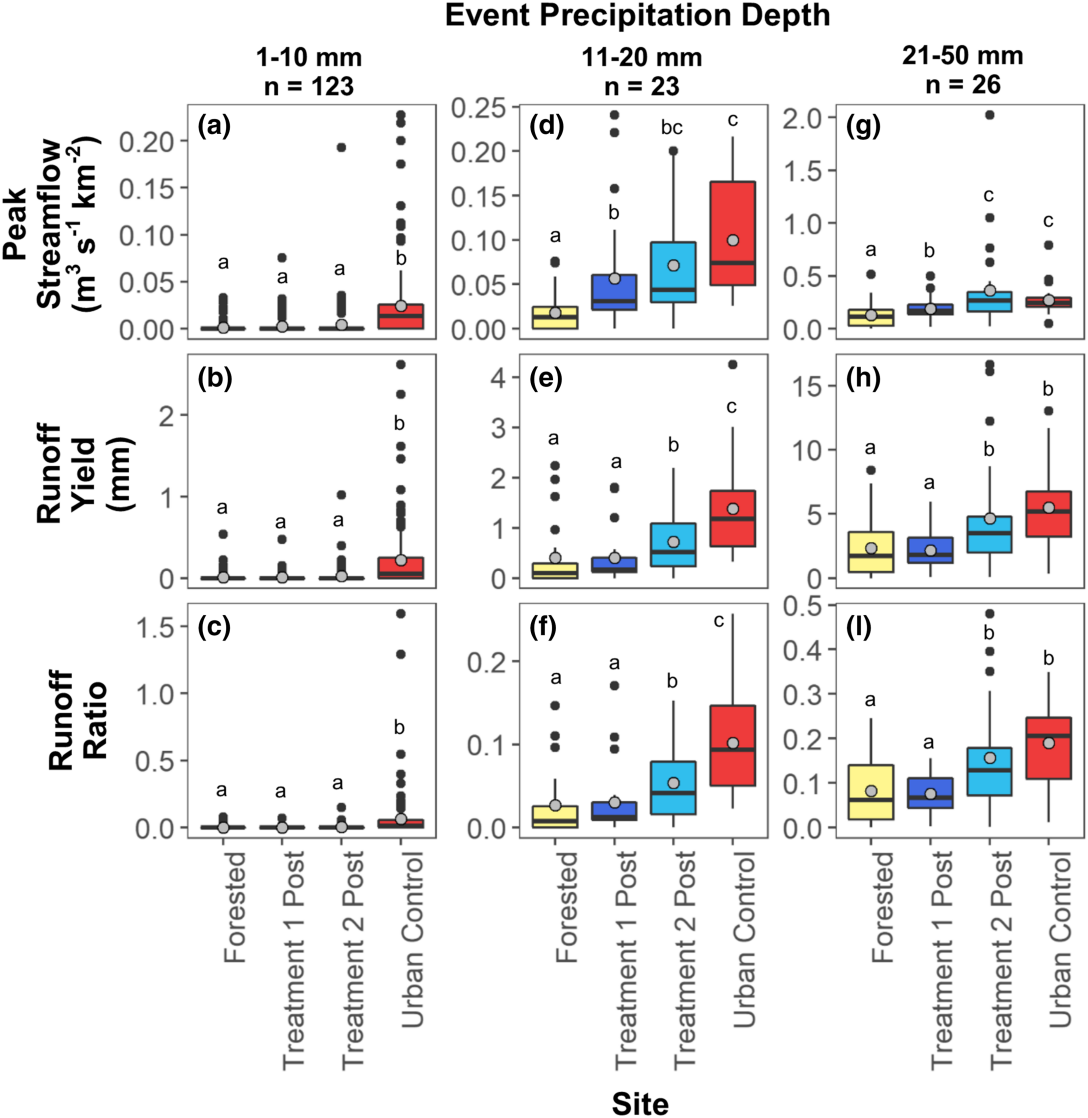
Boxplots of matched streamflow events during the after‐development period (2015–2018) in the treatment watersheds and the control watersheds. Streamflow metrics included peak streamflow normalized by area, runoff yield, and runoff ratio for three precipitation event categories. Boxplot box bounds the 25% and 75% quantiles, solid line shows the median, and gray circle shows the mean. Lower case letters show significant differences between sites based on pairwise comparisons using Wilcoxon rank sum test (*p* < .05). Note the *y*‐axes are different across precipitation categories because of the different magnitude of streamflow responses

For the smallest precipitation‐event category (1–10 mm), the urban control had significantly higher (*p* < .05) peak streamflow, runoff yield, and runoff ratios than the urban treatments and the forested site (Figure 7). The median measurement at the urban control for the three streamflow metrics was one to two orders of magnitude greater than the median for the three other sites (Table 5).

**Table 5:** Event statistics for peak streamflow, runoff yield, and runoff ratios for three precipitation‐event categories for matched‐streamflow events after watershed development. IQR indicates the interquartile range. Includes all matched events from 2015–2018

		Peak streamflow (m^3^ s^‐1^ km^‐2^)			Runoff Yield (mm)			Runoff Ratio (Runoff/Precipitation)	
Site	*N*	Mean	Median	IQR	Mean	Median	IQR	Mean	Median	IQR
Event Precipitation: 1–10 mm									
Forested	123	0.001	0.000	0.000	0.011	0.000	0.000	0.002	0.000	0.000
Urban Treatment 1 Post‐Development	123	0.002	0.000	0.000	0.010	0.000	0.000	0.001	0.000	0.000
Urban Treatment 2 Post‐Development	123	0.004	0.000	0.000	0.024	0.000	0.000	0.004	0.000	0.000
Urban Control	123	0.024	0.014	0.026	0.221	0.061	0.255	0.064	0.016	0.054
Event Precipitation: 11–20 mm									
Forested	23	0.018	0.013	0.025	0.400	0.102	0.292	0.027	0.008	0.026
Urban Treatment 1 Post‐Development	23	0.057	0.031	0.039	0.401	0.168	0.288	0.030	0.012	0.021
Urban Treatment 2 Post‐Development	23	0.071	0.044	0.068	0.724	0.516	0.846	0.054	0.041	0.063
Urban Control	23	0.102	0.074	0.118	1.428	1.216	1.105	0.104	0.113	0.093
Event Precipitation: 20–50 mm									
Forested	26	0.131	0.112	0.152	2.337	1.702	3.115	0.082	0.062	0.122
Urban Treatment 1 Post‐Development	26	0.189	0.170	0.096	2.159	1.816	1.934	0.076	0.067	0.066
Urban Treatment 2 Post‐Development	26	0.365	0.265	0.183	4.657	3.519	2.771	0.157	0.129	0.107
Urban Control	26	0.273	0.244	0.083	5.490	5.186	3.497	0.190	0.205	0.137

For medium precipitation depths (11–20 mm), peak streamflow at the forested site was significantly less (*p* < .05) than all other sites (Figure 7D). Peak streamflow for medium events was not significantly different (*p* > .05) between the urban treatments, but Urban Treatment 1 post‐development had lower peak streamflow than the urban control, whereas Urban Treatment 2 post‐development was similar to the urban control (Figure 7D). Median peak streamflow for medium events was 2.4 and 3.4 times greater in Urban Treatment 1 and 2 post‐development relative to the forested site, respectively (Table 5). Runoff yield and runoff ratios for the forested site and Urban Treatment 1 were not significantly different (*p* > .05) but were significantly lower than Urban Treatment 2 and the urban control (*p* < .05) for medium events (Figures 7E and 7F). Median runoff yield at Urban Treatment 2 was five times greater than the median at the forested site and the median runoff yield at the urban control was seven times greater than the median at Urban Treatment 1 (Table 5).

For the large precipitation‐event category (21–50 mm), Urban Treatment 1 post‐development was similar to the forested control, whereas Urban Treatment 2 post‐development was not significantly different from the urban control for any streamflow metric (Figures 7G‐7I). Urban Treatment 1 runoff yield and runoff ratios were not significantly different from the forested site, but median peak streamflow was 1.5 times greater at Urban Treatment 1 compared with the forested site (Table 5). Median peak streamflow, runoff yield, and runoff ratio at Treatment 2 was 1.6, 1.9, and 1.9 times greater than the median at Urban Treatment 1, respectively (Table 5).

### 3.5 Differences in streamflow magnitude and timing between Treatment watersheds

**Figure 8:**
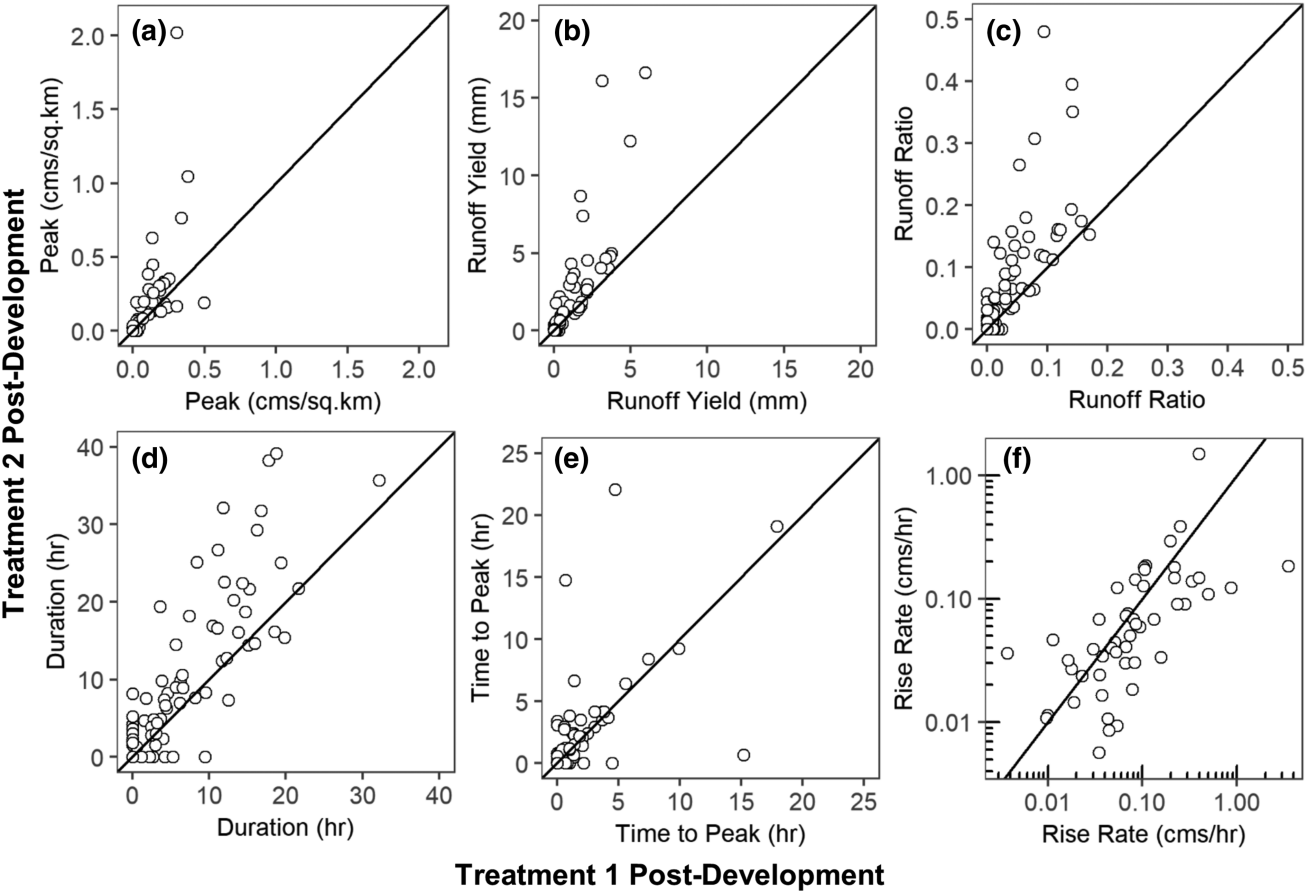
Differences in streamflow metrics for matched events in Urban Treatments 1 and 2 after watershed development. Solid lines indicate the 1:1 line where response was the same for Urban Treatments 1 and 2. Points below the line indicate greater streamflow response in Treatment 1. Points above the line indicate greater streamflow response in Urban Treatment 2

Streamflow metrics were compared for matched stormflow responses post‐development in Urban Treatment 1 and 2. This time period included 172 precipitation events, with detectable streamflow response occurring during 61 (35%) and 57 (33%) of those events for Urban Treatment 2 and Urban Treatment 1, respectively. The magnitude of streamflow response in Urban Treatment 2 was typically larger than Urban Treatment 1, with higher peak streamflow, runoff yield, and runoff ratio, longer duration events, longer time to peak, and shorter rise rates in Urban Treatment 2 (Figure 8). Peak streamflow, runoff yield, and runoff ratios were greater in Urban Treatment 2 than Urban Treatment 1 in 86%, 95%, and 95% of events with a detectable streamflow response, respectively. Duration and time to peak streamflow were greater in Urban Treatment 2 than Urban Treatment 1 in 89% and 77% of events with a detectable streamflow response, respectively (Figures 8D‐8E). Rise rates tended to be similar or slightly greater in Urban Treatment 1 compared with Urban Treatment 2 (Figure 8F).

### 3.6 Streamflow change in Treatment 2 before and after development

**Figure 9:**
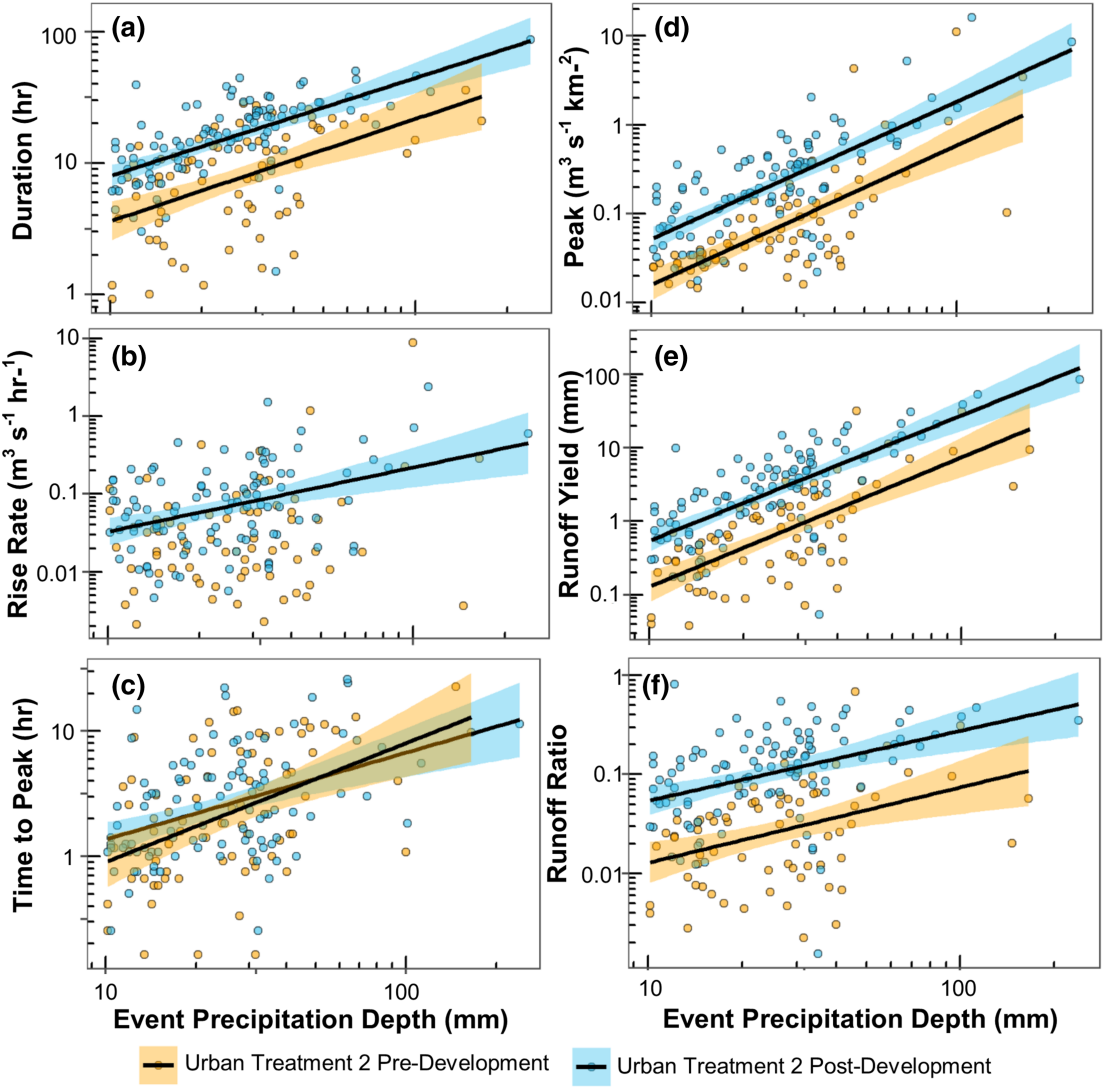
Log‐log linear regression for event streamflow timing (A‐C) and magnitude (D‐F) variables versus event precipitation depth for the Treatment 2 before and after. Only significant linear regressions are shown (*p* < .05). Shaded area around linear fit displays 95% confidence interval

Linear regression was used to compare the relation between precipitation depth and streamflow magnitude and timing variables in Urban Treatment 2 pre‐development and post‐development. Only precipitation events with greater than 10 mm were compared to examine responses above the breakpoint. Peak streamflow, runoff yield, runoff ratio, and event duration all increased relative to precipitation depth in Urban Treatment 2 post‐development compared with Urban Treatment 2 pre‐development (Figure 9). Regression models indicated greater model intercepts but no significant difference in model slopes for peak streamflow, runoff yield, runoff ratio, and event duration (Table S4). Rise rate and time to peak were highly variable and there was no detectable change in these timing metrics relative to precipitation depth post‐development (Figures 9B‐9C).

## 4 DISCUSSION

Multiple lines of evidence were used to assess changes in streamflow magnitude and timing in urban treatment watersheds that underwent suburban development with a high density of infiltration‐focused SCMs. Results indicate that streamflow responses in Urban Treatments 1 and 2 were both altered by suburban development. SCMs implemented with a design of 0% effective impervious area, meaning all impervious area drains to a SCM, were not able to completely mitigate the hydrologic response that results from suburban development. Regarding metrics of streamflow magnitude, Urban Treatment 1 was more hydrologically similar to the forested control than the urban control (Figure 7). In contrast, Urban Treatment 2, which had a higher SCM density and more impervious cover than Urban Treatment 1, was more similar to the urban control than the forested control (Figure 7) and peak streamflow and runoff yield increased post‐development (Figure 9). SCMs in the urban treatment watersheds provide the most hydrologic benefit during events with precipitation depths of 20 mm or less, a similar depth to the design criteria for most of the SCMs (25.4 mm).

Results indicated better hydrologic performance from watersheds with a high density of infiltration‐focused SCMs (urban treatments) than a watershed with a low density of detention‐focused SCMs (urban control). Urban Treatment 2 has 4% more impervious cover than the urban control, yet the precipitation threshold was 2.3 times higher (11.5 mm compared with 5 mm) in Urban Treatment 2 compared with the urban control (Table 3), indicating greater storage capacity in the urban treatment watershed. Median peak flow and runoff yield for medium events were 59% and 42% lower, respectively, in Urban Treatment 2 compared with the urban control (Table 5). Use of infiltration‐focused SCMs implemented at a watershed‐scale can provide enhanced attenuation of peak flow and runoff volumes compared with centralized‐detention SCMs.

Although the implementation of SCMS to mitigate 100% of the impervious area did not eliminate the effect of urban development, SCMs in Urban Treatments 1 and 2 were able to mitigate some of the effects of increased levels of impervious cover, particularly for runoff yield and for events with precipitation depths less than 20 mm. For example, runoff yield in Urban Treatment 1 post‐development was not significantly different than the forested control for any of the precipitation event categories, even though Urban Treatment 1 has 33% impervious cover (Figure 7). Urban Treatment 2 peak streamflow and runoff yield were lower or similar to the urban control for all event categories even through Urban Treatment 2 had 4% more impervious cover than the urban control (Figure 7). Similarly, precipitation depths needed to initiate a streamflow response and breakpoints for piecewise linear regressions of peak streamflow also indicated that response in Urban Treatment 1 was more similar to the forested site and that Urban Treatment 2 hydrologic response was between the forested and urban control (Figures 4 and 5). SCMs were able to mitigate hydrologic effects for events with depths less than 20 mm, however, this event size is considerably lower than the 1‐year 24‐hour event (66 mm) design criteria for detention pond sizing (MDE, 2000). If the SCM design goal is to maintain pre‐development hydrologic conditions in the urban treatment watersheds for events larger than 25.4 mm, then additional SCM stormwater storage is likely necessary.

Streamflow response in Urban Treatment 1 was less altered by development than Urban Treatment 2. Urban Treatment 1 had lower peaks, runoff yield, and runoff ratios and longer time‐to‐peak and shorter duration events than Urban Treatment 2 (Figure 8). Differences in streamflow response between the treatment watersheds may be related to three main factors, differences in 1) amount of impervious cover, 2) type and location of SCMs, and 3) precipitation intensity and depth. Urban Treatment 2 has 1.3 times more impervious cover (44%) than Urban Treatment 1 (33%). An increase of 11% impervious cover would result in a larger volume of runoff to be mitigated by SCMs. To mitigate this additional impervious cover, SCM density in Urban Treatment 2 is more than double that of Urban Treatment 1 (Table 1). However, even with this increase of SCM density in Urban Treatment 2, the increase in imperviousness was not completely mitigated. This may be attributed to differences in SCM types implemented in the two treatment watersheds. The SCMs in Urban Treatment 1 are largely composed of underground recharge chambers and infiltration trenches. In contrast, the SCMs in Urban Treatment 2 are dominated by micro‐bioretention and tree boxes, which have a small footprint and therefore a smaller storage volume before runoff overflows (Table S2). Although Urban Treatment 2 has a greater density of SCMs, it may still have a lower SCM total storage volume than Urban Treatment 1. Some streets in Urban Treatment 1, but not Urban Treatment 2, also have vegetated swales rather than curb‐and‐gutter infrastructure, which Woznicki, Hondula, and Jarnagin (2018) found to reduce total runoff and peak runoff rates at the neighborhood scale (Figure S1). Because Urban Treatments 1 and 2 both have dry detention ponds as the last line of SCM treatment before runoff is discharged into the stream, we would expect similar peak streamflow mitigation because design standards for dry detention are similar between treatments. However, results indicated that peak streamflow was typically greater in Urban Treatment 2 compared with Urban Treatment 1 (Figure 8A), which could indicate greater overflow from upstream parts of the network in Urban Treatment 2. Differences in precipitation patterns across the two urban treatment watersheds could have also attributed to differences in runoff metrics between treatment watersheds.

Although SCM density was greater in Urban Treatment 2, this was not an indication of better performance for watershed‐scale hydrologic response. An area for future work is the development of planning design tools, beyond treated imperious area, that provide indicators for watershed‐scale hydrologic performance as site‐scale design and SCM density were not found to correspond with performance. Detailed information on SCM stormwater storage capacity in all three urban watersheds would also help in understanding how SCM sizing and storage varied between the urban watersheds in this study. This SCM information was not readily available at the time of our study and precludes us having a more detailed understanding of potential storage capacity.

### 4.1 Streamflow breakpoints versus precipitation thresholds

Two approaches were used to detect precipitation depths needed to initiate streamflow response: 1) logistic regression and 2) piecewise linear regression. Both approaches identified similar precipitation depth thresholds for each site, with 75% event depth probabilities for logistic regressions all within breakpoint confidence intervals for peak streamflow piecewise regressions (Tables 3 and 4). Peak flow piecewise regression breakpoints for the forested control (11 mm) in Loperfido et al. (2014) was within the 95% confidence interval of the breakpoint identified in our study (Table 4). The Clarksburg forested control site breakpoint was similar to a forested site in Charlotte, NC, which had a peak streamflow breakpoint of 12 mm, but the urban control had a higher breakpoint than an urban site with 54% impervious cover in Charlotte (Bell et al., 2016). The difference in breakpoints fits with the expectation that it would take a greater depth of precipitation to initiate a flow response in a forested watershed compared with an urban watershed.

The breakpoint Loperfido et al., 2014 reported for Urban Treatment 1 post‐development was substantially lower than our study, 9.5 mm compared with 16.5 mm. This difference in Urban Treatment 1 post‐development breakpoints could be associated with different time periods of analysis. Loperfido et al., 2014 analyzed a shorter time‐period spanning March 2011 through September 2012, whereas our analysis included more than 7 years spanning January 2011 through September 2018. Loperfido et al., 2014 used the daily maximum 5‐minute streamflow value, whereas our study used the maximum peak streamflow from each storm event. Daily maximum discharge would miss events with two peaks in one day and events with peaks two or more days in a row, whereas event‐based metrics would identify these situations as discrete events. Differences in breakpoint estimates between our study and Loperfido et al., 2014 suggest that event‐based peak streamflow metrics are more sensitive in urban watersheds than aggregated daily metrics.

### 4.2 Challenges for detecting streamflow change

There are some confounding factors inherent in empirical analyses of watershed‐scale response. The primary confounding factor in this study was that we assumed precipitation patterns were similar across the entire study area because only a single rain gage was used to represent precipitation metrics. Precipitation depth and intensity can vary substantially across a relatively small area, particularly for convective storms that are short in duration and high in intensity (Cristiano, ten Veldhuis, & van de Giesen, 2017; Smith et al., 2012). Advances in recording spatial patterns in precipitation depth and intensity, such as HydroNEXRAD radar rainfall, will allow for more accurate estimates of precipitation metrics and may reduce the variance associated with regressing streamflow metrics against precipitation depth. Our inability to capture spatial variability in precipitation patterns may also explain why precipitation depth was a better predictor of streamflow response than precipitation intensity. Precipitation intensity was tested as it was important in other urban watersheds (Smith, Smith, Baeck, Villarini, & Wright, 2013) and would be expected to indicate infiltration‐excess overland flow and lead to flash and pluvial flooding (Rosenzweig et al., 2018; Smith & Rodriguez, 2017). However, precipitation depth may be more reliably measured with a single rain gage across neighbouring watersheds than precipitation intensity, leading to the observed importance of precipitation depth in determining streamflow response.

Short data gaps (< 2 hours between measurements) in the instantaneous streamflow necessitated the use of interpolation methods to maximize the number of complete streamflow responses included in the analysis and events that could be considered matched for treatment‐control comparisons. Construction activities also complicated the identification and interpretation of streamflow responses. Although these data gaps and construction activities did not affect our findings, it was critical to develop a routine protocol for quality control of the instantaneous streamflow data used in this study. Better information on the storage characteristics of SCMs implemented in the treatment watersheds would be useful in further explaining differences among hydrologic responses in all three urban watersheds.

Although this study design is one of the most robust in the world for assessing the effects of urban development and SCM implementation, future study designs would benefit from the inclusion of more control watersheds for quantifying the variability in end‐member conditions (e.g., forested control or urban control). The inclusion of more treatment watersheds with variable SCM densities and impervious cover would also provide additional opportunities to disentangle the factors driving hydrologic performance between Urban Treatments 1 and 2.

## 5 CONCLUSIONS AND IMPLICATIONS

We examined 14 years of event‐based streamflow metrics using instantaneous discharge in two urban treatment watersheds with a high density of infiltration‐focused SCMs (> 100 SCMs/km^2^), a forested control watershed, and an urban control watershed with a low density of detention‐focused SCMs (47 SCMs/km^2^). Results indicated that a high density of infiltration‐focused SCMs can provide enhanced mitigation of peak flows and runoff volumes compared with a watershed with a low density of centralized‐detention based SCMs. However, a high density of infiltration‐focused SCMs was not able to replicate forested conditions across a range of precipitation depths. Streamflow magnitude and timing were altered in the two urban treatment watersheds, even with SCMs implemented to treat 100% of the impervious area. Streamflow change was more severe in Urban Treatment 2 than Urban Treatment 1, with streamflow magnitude metrics in Urban Treatment 2 similar to the urban control. This difference is likely because of greater impervious cover in Treatment 2 (44%) compared with Treatment 1 (33%) which led to larger runoff volumes for SCMs to mitigate. Once SCM storage capacity is exceeded, runoff either bypasses SCMs or passes through with minimal treatment. Although streamflow changes were observed in both treatment watersheds, SCMs were able to mitigate some of the impacts of increased impervious cover particularly for events with depths less than 20 mm (0.8 inch). This is a considerably lower precipitation depth than the design storm used for SCM dry pond sizing, which is a 1‐year 24‐hour event equivalent to 2.6 inches (66 mm) of precipitation (MDE, 2000). Results suggest that additional SCMs are needed to maintain pre‐development hydrologic conditions in new suburban developments for precipitation events larger than 1 inch (25.4 mm). Long‐term monitoring studies that include high‐frequency measurements are essential for accurately understanding hydrologic changes as watersheds undergo land use change and for optimizing the design and installation of SCMs in urban and suburban settings.

## 6 DISCLAIMERS

Any use of trade, firm, or product names is for descriptive purposes only and does not imply endorsement by the U.S. Government. Although this work was reviewed by USEPA and approved for publication, it may not necessarily reflect official Agency policy.

## Supporting information

Table S1. Precipitation totals by water year and the deviation from 20‐year normal (negative drier, positive wetter). Data obtained from the National Climate Data Center precipitation gage at Damascus (USC00182336), with missing daily precipitation data filled with daily data from Washington Dulles International Airport (USW00093738).
Table S2. Number of each stormwater control measure type in each study watershed. Data represent SCMs installed as of February 2017.
Table S3. Housing type in each study watershed.
Table S4. Linear regression model estimates for streamflow timing and magnitude variables versus event precipitation depth for Urban Treatment 2. All predictor and response variables were log_10_ transformed.

Figure S1. Location and types for stormwater control measures in Urban Treatments 1 and 2.
