# Supplementary material for: Changes in event‐based streamflow magnitude and timing after suburban development with infiltration‐based stormwater management
Source: Hydrol Process. 2019 Nov 13;34(2):387–403. doi: 10.1002/hyp.13593 (PMC7006812; doi:10.1002/hyp.13593)
Supplement: Supplementary file 1 — Table S1. Precipitation totals by water year and the deviation from 20‐year normal (negative drier, positive wetter). Data obtained from the National Climate Data Center precipitation gage at Damascus (USC00182336), with missing daily precipitation data filled with daily data from Washington Dulles International Airport (USW00093738). Table S2. Number of each stormwater control measure type in each study watershed. Data represent SCMs installed as of February 2017. Table S3. Housing type in each study watershed. Table S4. Linear regression model estimates for streamflow timing and magnitude variables versus event precipitation depth for Urban Treatment 2. All predictor and response variables were log10 transformed. [file HYP-34-387-s001.docx]

Changes in event-based streamflow magnitude and timing after suburban development with infiltration-based stormwater management

**Supporting Information**

Table S1. Precipitation totals by water year and the deviation from 20-year normal (negative drier, positive wetter). Data obtained from the National Climate Data Center precipitation gage at Damascus (USC00182336), with missing daily precipitation data filled with daily data from Washington Dulles International Airport (USW00093738).

| Water year | Precipitation (mm) | Deviation from 20-year normal (mm) |
| --- | --- | --- |
| 2005 | 987 | -191 |
| 2006 | 1201 | 23 |
| 2007 | 1020 | -158 |
| 2008 | 1170 | -8 |
| 2009 | 1082 | -96 |
| 2010 | 1340 | 162 |
| 2011 | 1331 | 153 |
| 2012 | 1180 | 2 |
| 2013 | 1281 | 103 |
| 2014 | 1442 | 264 |
| 2015 | 1165 | -13 |
| 2016 | 1341 | 163 |
| 2017 | 990 | -188 |
| 2018 | 1435 | 257 |
| Monitoring period mean (2005-2018) | 1212 | 34 |
| 20-year normal (1981-2010) | 1178 | NA |

Table S2. Number of each stormwater control measure type in each study watershed. Data represent SCMs installed as of February 2017.

| SCM Type | Urban Control | Forested | Urban Treatment 1 | Urban Treatment 2 |
| --- | --- | --- | --- | --- |
| Oil and grit separator | 12 | 1 | 15 | 38 |
| Bioretention, quality control | 24 | -- | 8 | 1 |
| Bioswale | 1 | 1 | 2 | -- |
| Dry well connected to downspouts | -- | -- | 10 | 4 |
| Infiltration trench or berm | 6 | 1 | 1 | -- |
| Infiltration trench, underground | 4 | -- | 11 | 43 |
| Micro-Bioretention | 57 | 1 | -- | 24 |
| Oil/grit separator and sand filter | 1 | -- | -- | -- |
| Dry pond | 3 | -- | 3 | 5 |
| Dry pond, extended detention | 1 | -- | 4 | -- |
| Dry pond, sand filter base | 1 | -- | 1 | -- |
| Wet pond, extended detention | 1 | -- | -- | -- |
| Pond-wetland, extended detention | 3 | 1 | -- | -- |
| Porous pavement | -- | -- | 1 | -- |
| Sand filter, aboveground | 12 | -- | 12 | 11 |
| Stormceptor | 6 | -- | 3 | -- |
| Stormfilter | 7 | -- | 6 | 20 |
| Tree Box Filter | -- | -- | -- | 50 |
| Underground detention basin | 4 | -- | 43 | 19 |
| Vegetated/Grass Swale (km) | 1 | -- | 6 (5.2 km) | 4 (1.8 km) |
| Total Number of SCMs | 144 | 5 | 126 | 219 |

Table S3. Housing type in each study watershed.

| Watershed Name | Total Housing Parcel Density no/km^2^ | Percentage Single Family Attached | Percentage Single Family Detached |
| --- | --- | --- | --- |
| Forest Control | 4  (n = 14) | 0% | 100% |
| Urban Control | 144  (n = 446) | 41% | 59% |
| Urban Treatment 1 | 567  (n = 680) | 9% | 91% |
| Urban Treatment 2 | 805  (n = 644) | 50% | 50% |

Table S4. Linear regression model estimates for streamflow timing and magnitude variables versus event precipitation depth for Urban Treatment 2. All predictor and response variables were log_10_ transformed.

| Streamflow Metric | Treatment 2 Time Period | Intercept  (95% CI) | Slope  (95% CI) | R^2^ | p |
| --- | --- | --- | --- | --- | --- |
| Duration | Pre | -0.22 (-0.60 - 0.14) | 0.78 (0.52 - 1.03) | 0.26 | < 0.001 |
|  | Post | 0.16 (-0.05 - 0.36) | 0.74 (0.60 - 0.89) | 0.41 | <0.001 |
| Rise Rate | Pre | NA | NA | 0.04 | 0.052 |
|  | Post | -2.31 (-2.77 - -1.86) | 0.83 (0.51 - 1.15) | 0.15 | < 0.05 |
| Time to Peak | Pre | -0.99 (-1.50 - -0.49) | 0.95 (0.60 - 1.30) | 0.21 | <0.001 |
|  | Post | -0.56 (-0.90 - -0.21) | 0.69 (0.45 - 0.94) | 0.17 | <0.001 |
| Peak | Pre | -3.34 (-3.88 - -2.85) | 1.57 (1.21 - 1.92) | 0.51 | <0.001 |
|  | Post | -2.84 (-3.25 - -2.43) | 1.55 (1.26 - 1.84) | 0.52 | <0.001 |
| Runoff Yield | Pre | -2.65 (-3.26 - -2.05) | 1.76 (1.34 - 2.18) | 0.49 | <0.001 |
|  | Post | -1.97 (-2.42 - -1.53) | 1.71 (1.39 - 2.02) | 0.53 | <0.001 |
| Runoff Ratio | Pre | -2.65 (-3.26 - -2.05) | 0.76 (0.34 - 1.18) | 0.14 | <0.001 |
|  | Post | -1.97 (-2.42- -1.53) | 0.71 (0.39 - 1.02) | 0.16 | <0.001 |

**Supporting Figure**


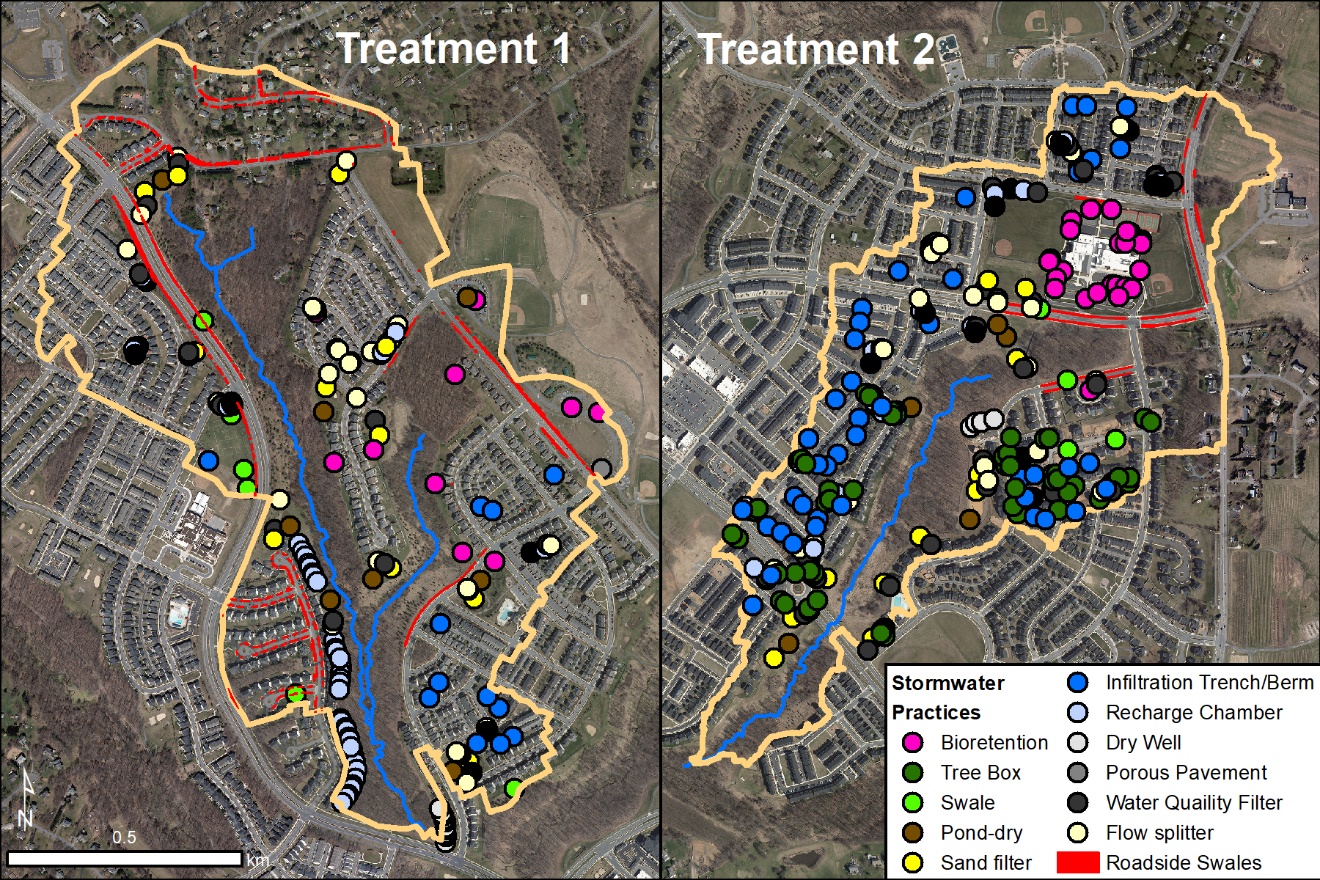


Figure S1. Location and types for stormwater control measures in Urban Treatments 1 and 2.
