# Supplementary figures and images for: Changes in event‐based streamflow magnitude and timing after suburban development with infiltration‐based stormwater management
Source: Hydrol Process. 2019 Nov 13;34(2):387–403. doi: 10.1002/hyp.13593 (PMC7006812; doi:10.1002/hyp.13593)

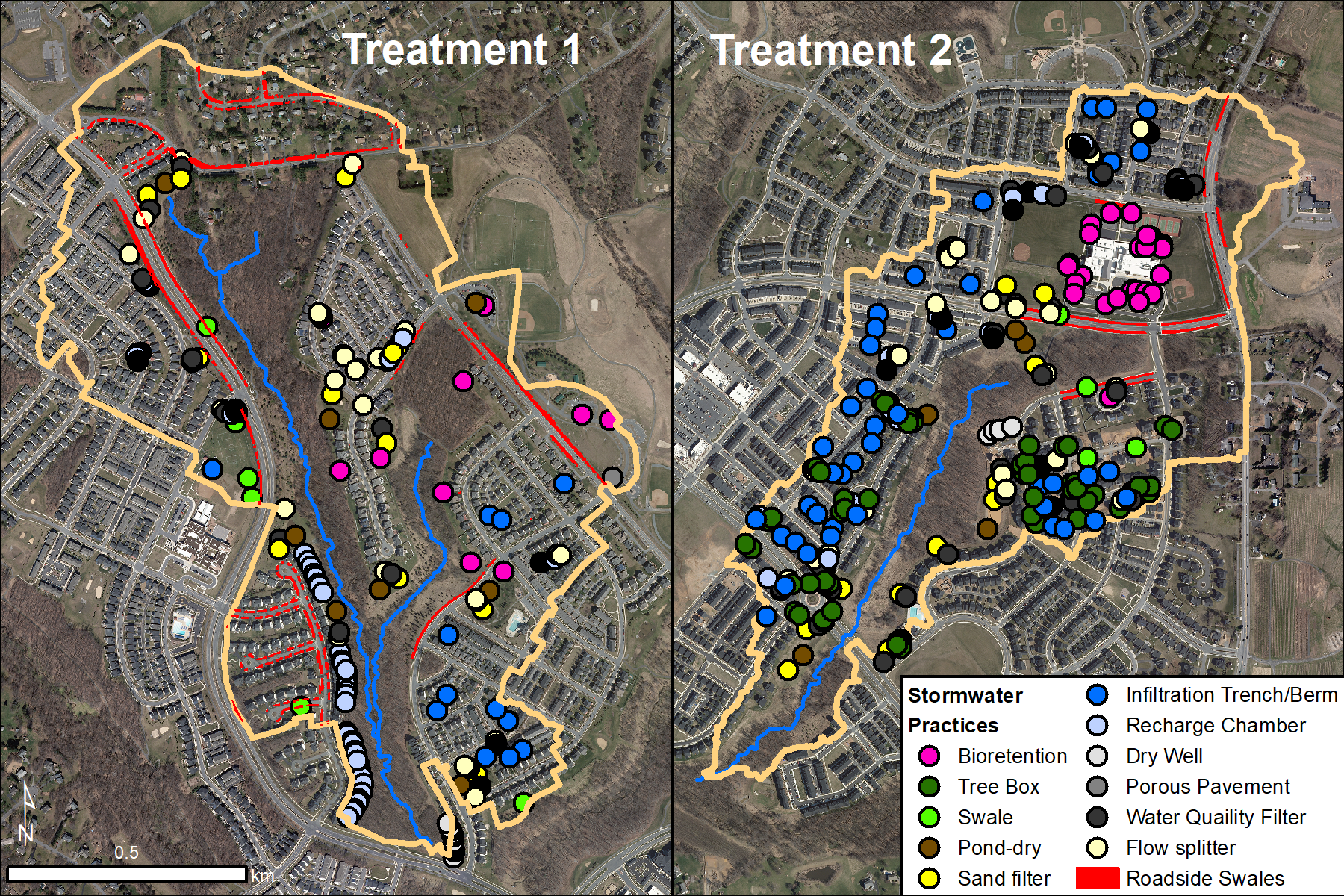

Supplement: Supplementary file 2 — Figure S1. Location and types for stormwater control measures in Urban Treatments 1 and 2. [file HYP-34-387-s002.tif]
